# An Improved Scheduling Algorithm for Data Transmission in Ultrasonic Phased Arrays with Multi-Group Ultrasonic Sensors

**DOI:** 10.3390/s17102355

**Published:** 2017-10-16

**Authors:** Wenming Tang, Guixiong Liu, Yuzhong Li, Daji Tan

**Affiliations:** 1 School of Mechanical & Automotive Engineering, South China University of Technology, Guangzhou 510641, China; tang.wm@mail.scut.edu.cn (W.T.); JDXlyz@hzcollege.com (Y.L.); 2 Guangzhou Doppler Electronic Technologies Co., Ltd., Guangzhou 510663, China; tandaji@cndoppler.cn

**Keywords:** ultrasonic phased array, scheduling algorithm, FIFOs, multi-group sensors, FPGA, bandwidth utilization

## Abstract

High data transmission efficiency is a key requirement for an ultrasonic phased array with multi-group ultrasonic sensors. Here, a novel FIFOs scheduling algorithm was proposed and the data transmission efficiency with hardware technology was improved. This algorithm includes FIFOs as caches for the ultrasonic scanning data obtained from the sensors with the output data in a bandwidth-sharing way, on the basis of which an optimal length ratio of all the FIFOs is achieved, allowing the reading operations to be switched among all the FIFOs without time slot waiting. Therefore, this algorithm enhances the utilization ratio of the reading bandwidth resources so as to obtain higher efficiency than the traditional scheduling algorithms. The reliability and validity of the algorithm are substantiated after its implementation in the field programmable gate array (FPGA) technology, and the bandwidth utilization ratio and the real-time performance of the ultrasonic phased array are enhanced.

## 1. Introduction

The technology of multi-group ultrasonic sensors that consist of lots of piezoelectric elements and various scanning patterns of an ultrasonic phased array (UPA) have recently attracted widespread attention in the non-destructive testing area [1,2]. The UPA produces a series of the ultrasonic waves controlled by the amplitudes and phases of the electrical pulses to excite a series of elements of the sensors. The waves can easily penetrate inside some materials by adjusting their radiation direction to synthesize flexible and rapidly focused scanning ultrasonic beams. The parameters of beams such as angles, focal distances, and focal spot sizes can be readily tuned with suitable software. Therefore, the beams can be used to detect defects that possibly occur at random positions of the materials [3,4,5,6].

To increase the focusing ability, a UPA instrument is often equipped with multiple ultrasonic sensors to collect the ultrasonic echo data from different directions. Each sensor can work in one or more groups so that a variety of scanning modes are generated [7,8,9,10], which can be called as a multi-group scanning, and each group scanning includes many focused beams. Hence, the number of the sensors and the scanning groups are two important factors to determine detection accuracy [11,12], such as size, location, and orientation of defects. For example, Song et al. verified that a large-aperture hemispherical phased array can restore a sharp focus and maximize acoustic energy delivery at target tissue [11]. Regardless of the orientation of individual focused beams, the multiple focused beams can change their focal depths and sweeping angles through the phase interference. As a consequence, it is possible to precisely detect the position and the size of defects by means of increasing the number of the sensors, the scanning groups, and the focused beams. However, this strategy will in turn significantly increase the amount of scanning data in the process of the defect detection, which makes these data difficult to be transmitted to a peripheral through a single (or small quantity) high-speed serial bus, and subsequently produces an ultrasound image.

Each focused beam often brings different sampling rates and sizes of data stream. During the transmission process, different data streams compete against each other to gain access to the unique high-speed serial bus. An excellent transmission scheduling algorithm should allow all the data streams to be transferred to a peripheral without any blocking in a serialized way. Otherwise, the data streams would be blocked or severely delayed. Therefore, it is very desirable to design an effective algorithm to serially transmit a great amount of the data streams. Several well-known scheduling algorithms have been proposed, such as Time Division Multiple Access (TDMA) [13] and Round Robin (RR) [14]. The verification, analysis, and comparison of the two algorithms were presented in literature [15], which proves that the TDMA strategy based on the fixed allocation of a time slot to each master process may lead to important latencies as a time slot, and the RR protocol allows any unused slots to be reallocated to a master process to provide higher bandwidth. Unfortunately, the process of the reallocation will make the time slice resources more fragmented, and increase the complexity of the scheduling algorithm. Multiple examples of implementation for the scheduling algorithm are available in the open literatures [16,17,18,19,20,21,22,23]. Srinivasan et al. designed a self-configuring scheduling protocol for ultrasonic sensor systems by using an algorithm of the timeslot allocation, which simplified the deployment of the present detection system [16]. Long et al. proposed a time-division-multiple-access-based energy consumption balancing algorithm for the general k-hop wireless sensor networks, where one data packet is collected in one cycle, and the results demonstrated the effectiveness of the algorithm in terms of energy efficiency and time slot scheduling [19]. However, although these strategies can effectively improve the efficiency of the data transmission, they increase the complexities of both hardware and software, and their application scopes are limited, which makes such strategies not suitable for UPA of the multi-group sensor scanning system because of limited hardware and software resources and high real-time request.

FPGA, which is short for the term field programmable logic gate array, has the characteristics of static system repeatable programming and dynamic system reconfiguration, so that hardware can be modified programmatically, and FPGA also is a special kind of ASIC with the advantages of parallel processing, high speed and flexibility. In this paper, we used a series of FIFOs as high-speed caches and cache times as weights to propose a novel FIFOs and bandwidth-sharing scheduling (MFBSS) algorithm of the data transmission, where the lengths of the FIFOs are achieved by a series of multivariate equations. Actually, the algorithm shows many advantages such as real-time and high efficiency when it is implemented by FPGA technology. As far as the UPA system of the array sensors is concerned, we designed a data stream transmission scheduling mode based on the MFBSS algorithm, with which reading operations among all the FIFOs shares a fast reading bus without time slot waiting when the reading bus switches between any two FIFOs. Hence, such algorithm gives the maximum bandwidth utilization ratio and improves the real-time performance of the UPA instruments with minimal consumption of time and space resources.

In Section 2 of the paper, we will describe the data transmission of ultrasonic scanning for UPA [24,25,26]. In Section 3, we will study scheduling mechanism of the MFBSS algorithm for the data transmission. Section 4 will describe the results of implementation for the scheduling algorithm by FPGA technology. Finally, Section 5 will summarize the research to derive the conclusion.

## 2. Multi-Group Sensor Scanning Ultrasonic Data Transmission

**Figure 1:**
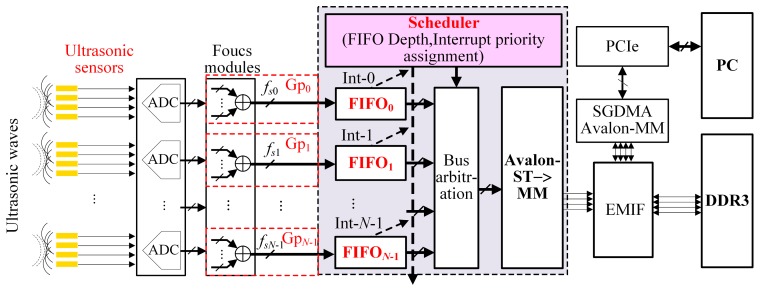
The diagram of the ultrasonic data transmission for the multi-sensor scanning.

Figure 1 shows the UPA data transmission framework of the bandwidth-sharing with multiple scanning patterns [7,8,9,10]. In order to realize the optimal sampling of the UPA’s echoes, different frequency echoes should be digitized with different sampling frequencies [27,28,29]. A sensor with a frequency of *f_p_* Hz produces ultrasonic echoes with the same frequency after excitation, and thus the sampling frequency is *f*_s_ = K × *f_p_* Hz (K is a scaling factor, and K ≥ 2). Hence, *N-*group sensors can form *N*-group scanning patterns, generating *N* sampling frequencies (*f_s_*_0_ ~ *f_sN_*_−1_, where 0 and *N* − 1 represent the numbers of sampling) and forming *N* focusing beams with specific speeds and sizes.

As shown in Figure 1, the data of various scanning groups such as Gp_0_, Gp_1_, …, and Gp*_N_*_−1_ produced from the ultrasonic sensors are written into FIFO_0_, FIFO_1_, …, FIFO*_N_*_−1_, respectively, which are cached by a DDR3 through the Avalon bus in the bandwidth-sharing way [30]. Then, the data from the DDR3 are transmitted to the host computer through the PCIe bus [31,32]. The entire data transmission process is controlled by a bandwidth scheduler, which is composed of a controller with all the FIFOs’ lengths and a reading arbiter, and usually runs the following scheduling algorithms such as First Come First Serve (FCFS), TDMA and Equal Time Slice Polling Scheduling (ETSPS) based on the principle of the RR scheduling which will be mentioned in Section 4, and so on. This paper will adopt the MFBSS algorithm to realize reading operations from every FIFO without time slot waiting through adjusting the lengths of FIFOs, timings of the reading and writing, and priority of the interrupts. Therefore, this algorithm can not only ensure the data transmission synchronization but also maximize the bandwidth utilization in all groups, which is readily implemented by FPGA technology with parallel processing.

## 3. Data Transmission Scheduling Mechanism of MFBSS Algorithm

### 3.1. The principle of the Maximal Bandwidth Utilization

To evaluate the utilization ratio of the data transmission bandwidth of the *N*-group scanning in the multi-input and single-output interfaces of the UPA system, the following requirements are satisfied:

- Data transmission models Gp(*n*), *n* = 0, 1, …, *N* − 1 are independent from each other and have identical distributions for every group.
- The sum of the data bandwidth [$\sum_{n=0}^{N-1}B_{v-Gp}(n)$ ] of all the groups and the sum of the memory bandwidth ($\sum B_{v-RAM}$) and the sum of the transmission bandwidth ($\sum B_{v-Trans}$) of the peripheral need to satisfy the following inequality:
  (1) $$\sum_{n=0}^{N-1}B_{v-Gp}(n)\leq \min(\sum B_{v-RAM},\sum B_{v-Trans})$$

The defined parameters of the *N*-group scanning and the *N* FIFOs caches are listed in Table 1. The writing bandwidth and the reading bandwidth of the *n*th FIFO*_n_* are *V_W_*(*n*) [*V_W_*(*n*) = *f_sn_* × ΔB] and *V_R_* bit/s, respectively. The sum of the writing bandwidth of all the FIFOs [$\sum_{n=0}^{N-1}V_{W}(n)$] should equal to the sum of the transmission bandwidth of the *N*-group scanning data [$\sum_{n=0}^{N-1}B_{v-Gp}(n)$], i.e., $\sum_{n=0}^{N-1}V_{W}(n)$ = $\sum_{n=0}^{N-1}B_{v-Gp}(n)$. Likewise, the sum of the reading bandwidth (*V_R_*) of all the FIFOs should equal to the sum of the transmission bandwidth of the DDR3 bandwidth ($\sum B_{v-RAM}$), i.e., *V_R_* =$\sum B_{v-RAM}$. When the Equation (1) becomes an equality, the maximum bandwidth utilization ratio is achieved, i.e., the single-output bandwidth equals to the sum of the multi-input bandwidths from the FIFOs. Consequently, the mathematical principle of the maximal bandwidth utilization ratio can be written as Equation (2).

(2) $$\left\{ \begin{array}{l} \sum_{n=0}^{N-1}B_{v-Gp}(n)\leq \sum B_{v-RAM}\\ \sum_{n=0}^{N-1}B_{v-Gp}(n)=\sum_{n=0}^{N-1}V_{W}(n)\\ \sum B_{v-RAM}=V_{R} \end{array}\right.\Rightarrow V_{R}=\sum_{n=0}^{N-1}V_{W}(n)$$

### 3.2. Realization of the Maximal Bandwidth Utilization Ratio

According to Equation (2), the mathematical model of the *N* FIFOs’ length functions of *L*(*i*), *i* = 0, 1, …, *N* − 1, [*L*(0) ≤ *L*(1) ≤ … ≤ *L*(*N* − 1), FIFO_0_, FIFO_1_, …, FIFO*_N_*_−1_] can be described as follows:

- Assuming that at the moment $T_{i}^{j}$, when the FIFO*_i_* is read until empty, the reading operation of the FIFO*_i_* will be disabled.
- At the next $T_{i}^{j+1}$, when the FIFO*_i_* is full and the amount of the data is *L*(*i*) (*i* = 0, 1, …, *N* − 1), the reading operation of the FIFO*_i_* will be enabled.

**Figure 2:**
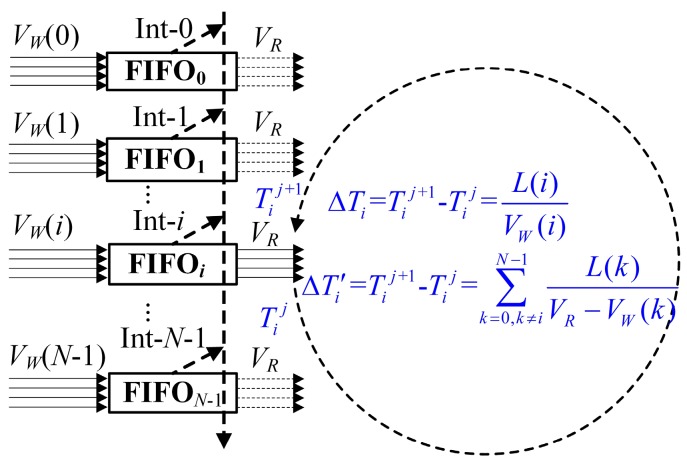
The time slot transition diagram of the *N* FIFOs reading operations.

When the FIFO*_i_* transfers from empty to full (where the consumed time is Δ*T_i_* = $T_{i}^{j+1}$ − $T_{i}^{j}=\frac{L(i)}{V_{W}(i)}$ and a reading interrupt is produced), the FIFO*_i_* will gain access to the reading of the Avalon bus. During this process, the other FIFOs with the number of 0, 1, …, *i* + 1, *i* + 2, …, *N* − 1 have also transferred from full to empty with the consumed time of Δ*T'_i_* = $\sum_{k=0,k\neq i}^{N-1}\frac{L(k)}{V_{R}-V_{W}(k)}$. The time slot transition diagram of the *N* FIFOs reading operations is shown in Figure 2. Because Δ*T_i_* = Δ*T'_i_*, i.e., Δ*T'_i_* − Δ*T_i_* = 0, *i* = 0, 1, …, *N* − 1, the mathematical equations of the N FIFOs’ length functions of *L*(*i*), *i* = 0, 1, …, *N* − 1 can be easily described in Equation (3).

(3) $$\left\{ \begin{array}{l} -\frac{L\left(0\right)}{V_{W}\left(0\right)}+\frac{L(1)}{V_{R}-V_{W}(1)}+\frac{L(2)}{V_{R}-V_{W}(2)}+\cdots +\frac{L(N-1)}{V_{R}-V_{W}(N-1)}=0\\ \frac{L(0)}{V_{R}-V_{W}(0)}-\frac{L\left(1\right)}{V_{W}\left(1\right)}+\frac{L(2)}{V_{R}-V_{W}(2)}+\cdots +\frac{L(N-1)}{V_{R}-V_{W}(N-1)}=0\\ \;\;\;\;\;\;\vdots \;\;\;\;\;\;\;\;\;\;\;\;\;\;\;\;\;\;\;\;\;\;\;\;\vdots \;\;\;\;\;\;\;\;\;\;\;\;\;\;\;\;\;\;\;\;\;\;\;\;\;\;\;\vdots \;\;\;\;\;\;\;\;\;\;\;\;\;\;\;\;\;\;\;\;\;\;\;\;\;\vdots \;\;\\ \frac{L(0)}{V_{R}-V_{W}(0)}+\frac{L(1)}{V_{R}-V_{W}(1)}+\cdots +\frac{L(N-2)}{V_{R}-V_{W}(N-2)}-\frac{L\left(N-1\right)}{V_{W}\left(N-1\right)}=0 \end{array}\right.$$

where *L*(*i*) ≠ 0, *i* = 0, 1, …, *N* − 1 in Equation (3). A series of new variables are given in Equation (4) for the simplification of Equation (3).

(4) $$\left\{ \begin{array}{l} K_{0}^{\prime }=\frac{1}{V_{W}(0)},\;\;\;\;\;\;\;\;K_{1}^{\prime }=\frac{1}{V_{W}(1)},\cdots ,K_{N-1}^{\prime }=\frac{1}{V_{W}(N-1)}\\ K_{0}=\frac{1}{V_{R}-V_{W}(0)},K_{1}=\frac{1}{V_{R}-V_{W}(1)},\cdots ,K_{N-1}=\frac{1}{V_{R}-V_{W}(N-1)}\\ V_{R}=\sum_{i=0}^{N-1}V_{W}(i) \end{array}\right.$$

Equation (3) is transformed into a matrix of Equation (5):

(5) $$\left(\begin{array}{cccc} -K_{0}^{\prime } & K_{1} & \cdots & K_{N-1}\\ K_{0} & -K_{1}^{\prime } & \cdots & K_{N-1}\\ \vdots & \vdots & \vdots & \vdots \\ K_{0} & K_{1} & \cdots & -K_{N-1}^{\prime } \end{array}\right)\left(\begin{array}{c} L(0)\\ L(1)\\ \vdots \\ L(N-1) \end{array}\right)=\left(\begin{array}{c} 0\\ 0\\ \vdots \\ 0 \end{array}\right)\overset{A=\left(\begin{array}{cccc} -K_{0}^{\prime } & K_{1} & \cdots & K_{N-1}\\ K_{0} & -K_{1}^{\prime } & \cdots & K_{N-1}\\ \vdots & \vdots & \vdots & \vdots \\ K_{0} & K_{1} & \cdots & -K_{N-1}^{\prime } \end{array}\right),\vec{L}=\left(\begin{array}{c} L(0)\\ L(1)\\ \vdots \\ L(N-1) \end{array}\right),\vec{0}=\left(\begin{array}{c} 0\\ 0\\ \vdots \\ 0 \end{array}\right)}{⇄}A\cdot \overset{⇀}{L}=\vec{0}$$

The matrix A is achieved by elementary row transformation, and then the triangular array is applied:

(6) $$A\sim \left(\begin{array}{ccccc} -K_{0}^{\prime } & K_{1} & \cdots & \cdots & K_{n-1}\\ K_{0}+K_{0}^{\prime } & -(K_{1}+K_{1}^{\prime }) & 0 & \cdots & 0\\ 0 & \vdots & \vdots & \vdots & \vdots \\ \vdots & \vdots & K_{N-3}+K_{N-3}^{\prime } & -(K_{1}+K_{N-2}^{\prime }) & 0\\ 0 & \cdots & 0 & K_{N-2}+K_{N-2}^{\prime } & -(K_{N-1}+K_{N-1}^{\prime }) \end{array}\right)\sim \left(\begin{array}{cccc} f_{K}(x_{0}) & K_{1} & \cdots & K_{N-1}\\ 0 & f_{K}(x_{1}) & \cdots & K_{N-1}\\ \vdots & \vdots & \vdots & \vdots \\ 0 & \cdots & 0 & f_{K}(x_{N-1}) \end{array}\right)$$

$f_{K}(x_{i+1})=\frac{K_{i+1}+K_{i+1}^{\prime }}{K_{i}+K_{i}^{\prime }}\cdot f_{K}(x_{i})+K_{i+1}$, *i* = 0, 1, …, *N* − 2, *f_K_*(*x*_0_) = −*K'*_0_, and $f_{K}(x_{i+1})$ can be done by using the following recursion:

$$\begin{array}{cc} f_{K}(x_{i+1}) & =\frac{K_{i+1}+K_{i+1}^{\prime }}{K_{i}+K_{i}^{\prime }}\cdot \left(\frac{K_{i}+K_{i}^{\prime }}{K_{i-1}+K_{i-1}^{\prime }}\cdot \left(\cdots \left(\frac{K_{1}+K_{1}^{\prime }}{K_{0}+K_{0}^{\prime }}\cdot f_{K}(x_{0})+K_{1}\right)+\cdots \right)+K_{i}\right)+K_{i+1}\\ & =\frac{K_{i+1}+K_{i+1}^{\prime }}{K_{i}+K_{i}^{\prime }}\cdot \frac{K_{i}+K_{i}^{\prime }}{K_{i-1}+K_{i-1}^{\prime }}\cdot \cdots \cdot \frac{K_{1}+K_{1}^{\prime }}{K_{0}+K_{0}^{\prime }}\cdot f_{K}(x_{0})+\frac{K_{i+1}+K_{i+1}^{\prime }}{K_{i}+K_{i}^{\prime }}\cdot \frac{K_{i}+K_{i}^{\prime }}{K_{i-1}+K_{i-1}^{\prime }}\cdot \cdots \cdot \frac{K_{2}+K_{2}^{\prime }}{K_{1}+K_{1}^{\prime }}\cdot K_{1}\\ & +\frac{K_{i+1}+K_{i+1}^{\prime }}{K_{i}+K_{i}^{\prime }}\cdot \frac{K_{i}+K_{i}^{\prime }}{K_{i-1}+K_{i-1}^{\prime }}\cdot \cdots \cdot \frac{K_{3}+K_{3}^{\prime }}{K_{2}+K_{2}^{\prime }}\cdot K_{2}+\cdots +\frac{K_{i+1}+K_{i+1}^{\prime }}{K_{i}+K_{i}^{\prime }}\cdot K_{i}+K_{i+1}\\ & =\frac{K_{i+1}+K_{i+1}^{\prime }}{K_{0}+K_{0}^{\prime }}\cdot f_{K}(x_{0})+\frac{K_{i+1}+K_{i+1}^{\prime }}{K_{1}+K_{1}^{\prime }}\cdot K_{1}+\frac{K_{i+1}+K_{i+1}^{\prime }}{K_{2}+K_{2}^{\prime }}\cdot K_{2}+\cdots +\frac{K_{i+1}+K_{i+1}^{\prime }}{K_{i+1}+K_{i+1}^{\prime }}\cdot K_{i+1}\\ & =\left(K_{i+1}+K_{i+1}^{\prime }\right)\cdot \left(\frac{K_{i+1}}{K_{i+1}+K_{i+1}^{\prime }}+\frac{K_{i}}{K_{i}+K_{i}^{\prime }}+\cdots +\frac{K_{2}}{K_{2}+K_{2}^{\prime }}+\frac{K_{1}}{K_{1}+K_{1}^{\prime }}-\frac{K_{0}^{\prime }}{K_{0}+K_{0}^{\prime }}\right)\\ & =\left(K_{i+1}+K_{i+1}^{\prime }\right)\cdot \left[\left(\sum_{j=1}^{i+1}\frac{K_{j}}{K_{j}+K_{j}^{\prime }}\right)-\frac{K_{0}^{\prime }}{K_{0}+K_{0}^{\prime }}\right] \end{array}$$

According to Equation (4), $f_{K}(x_{i+1})$ can be described as Equation (7).

(7) $$\begin{array}{cc} f_{K}(x_{i+1}) & =\left(\frac{1}{V_{R}-V_{W}(i+1)}+\frac{1}{V_{W}(i+1)}\right)\cdot \left(\sum_{j=1}^{i+1}\frac{\frac{1}{V_{R}-V_{W}(j)}}{\frac{1}{V_{R}-V_{W}(j)}+\frac{1}{V_{W}(j)}}-\frac{\frac{1}{V_{W}(0)}}{\frac{1}{V_{R}-V_{W}(0)}+\frac{1}{V_{W}(0)}}\right)\\ & =\frac{1}{\left(V_{R}-V_{W}(i+1)\right)\cdot V_{W}(i+1)}\cdot \left(\sum_{j=0}^{i+1}V_{W}(j)-V_{R}\right) \end{array}$$

For the *N*-group scanning of the UPA system, when *i* = *N*, according to the Equation (2), *V_R_* = $\sum_{n=0}^{N-1}V_{W}(n)$, and $f_{K}(x_{N-1})$ = ${\frac{1}{\left(V_{R}-V_{W}(N-1)\right)\cdot V_{W}(N-1)}\cdot \frac{1}{V_{R}}\cdot \left(\sum_{j=0}^{N-1}V_{W}(j)-V_{R}\right)|}_{V_{R}=\sum_{j=0}^{N-1}V_{W}(j)}=0$. The matrix A can be transformed to $A^{\prime }$ through the primary row transformation:

$$A=\left(\begin{array}{cccc} -K_{0}^{\prime } & K_{1} & \cdots & K_{N-1}\\ K_{0} & -K_{1}^{\prime } & \cdots & K_{N-1}\\ \vdots & \vdots & \vdots & \vdots \\ K_{0} & K_{1} & \cdots & -K_{N-1}^{\prime } \end{array}\right)\sim \left(\begin{array}{cccccc} f_{K}(x_{0}) & K_{1}-f_{K}(x_{1}) & 0 & 0 & \cdots & 0\\ 0 & f_{K}(x_{1}) & K_{2}-f_{K}(x_{2}) & 0 & \cdots & 0\\ \vdots & \vdots & \vdots & \vdots & \vdots & 0\\ 0 & \cdots & \cdots & 0 & f_{K}(x_{N-2}) & K_{2}-f_{K}(x_{N-1})\\ 0 & \cdots & \cdots & \cdots & 0 & f_{K}(x_{N-1}) \end{array}\right)\underline{\underline{f_{K}(x_{N-1})=0}}\left(\begin{array}{cccccc} f_{K}(x_{0}) & K_{1}-f_{K}(x_{1}) & 0 & 0 & \cdots & 0\\ 0 & f_{K}(x_{1}) & K_{2}-f_{K}(x_{2}) & 0 & \cdots & 0\\ \vdots & \vdots & \vdots & \vdots & \vdots & 0\\ 0 & \cdots & \cdots & 0 & f_{K}(x_{N-2}) & K_{2}\\ 0 & \cdots & \cdots & \cdots & 0 & 0 \end{array}\right)=A^{\prime }$$

Because the rank R(A) of the matrix A and the rank $R(A^{\prime })$ of the matrix $A^{\prime }$ have the following relation $R(A)=R(A^{\prime })<N$, Equation (5) has an infinite number of the solutions, and because $A\cdot \vec{L}=\vec{0}$⇔$A^{\prime }\cdot \vec{L}=\vec{0}$, and the solutions can be expressed as follows: 

*f_K_*(*x_i_*) × *L*(*i*) + (*K_i_*_+1_ − *f_K_*(*x_i_*_+1_)) × *L*(*i*+1) = 0, (*i* = 0, 1, …, *N* − 2), and *L*(*i*) = $\frac{f_{K}(x_{i+1})-K_{i+1}}{f_{K}(x_{i})}\cdot L(i+1),$ (*i* = 0, 1, …, *N* − 2), and *L*(*i*) can be further deduced forward:

(8) $$\begin{array}{cc} L(i)= & \frac{f_{K}(x_{i}{}_{+1})-K_{i+1}}{f_{K}(x_{i})}\cdot \frac{f_{K}(x_{i}{}_{+2})-K_{i+2}}{f_{K}(x_{i}{}_{+1})}\cdot \cdots \cdot \frac{f_{K}(x_{N-1})-K_{N-1}}{f_{K}(x_{N-2})}\cdot L(N-1)\\ & =\prod_{j=i}^{N-2}\frac{f_{K}(x_{j+1})-K_{j+1}}{f_{K}(x_{j})}\cdot L(N-1) \end{array}$$

Substituting the expression of *f_K_*(*x_i_*_+1_) from Equation (7) into Equation (8). The values of *L*(*i*), *i* = 0, 1, …, *N* − 1 are obtained, as shown in Equation (9):

(9) $$\left\{ \begin{array}{l} L(0)=\frac{\left(V_{R}-V_{W}(0)\right)\cdot V_{W}(0)}{\left(V_{R}-V_{W}(N-1)\right)\cdot V_{W}(N-1)}\cdot L(N-1)\\ \;\;\;\;\;\;\;\vdots \\ L(i)=\frac{\left(V_{R}-V_{W}(i)\right)\cdot V_{W}(i)}{\left(V_{R}-V_{W}(N-1)\right)\cdot V_{W}(N-1)}\cdot L(N-1)\\ \;\;\;\;\;\;\;\vdots \\ L(N-2)=\frac{\left(V_{R}-V_{W}(N-2)\right)\cdot V_{W}(N-2)}{\left(V_{R}-V_{W}(N-1)\right)\cdot V_{W}(N-1)}\cdot L(N-1)\\ L(N-1)=L(N-1) \end{array}\right.$$

when Equation (9) is multiplied by a term of $\frac{\left(V_{R}-V_{W}(N-1)\right)\cdot V_{W}(N-1)}{L(N-1)}$, a set of fundamental solutions $\vec{\xi }$ to the equations of $A\cdot \vec{L}=\vec{0}$ will be obtained:

$$\vec{\xi }={\left(\left(V_{R}-V_{W}(0)\right)\cdot V_{W}(0),\;\left(V_{R}-V_{W}(1)\right)\cdot V_{W}(1),\;\cdots ,\;\left(V_{R}-V_{W}(N-1)\right)\cdot V_{W}(N-1)\right)}^{T}.$$

Therefore, the solutions to the equations of $A\cdot \vec{L}=\vec{0}$ can be expressed as $\vec{L}=\alpha \cdot \vec{\xi }$ (*α*∈**R**^+^). The length function of *L*(*i*), *i* = 0, 1, …, *N* − 1 of the FIFOs has a proportional relation, as showed in Equation (10).

(10) $$\begin{array}{cc} L(0):L(1):\;\cdots :\;L(N-1)= & (V_{R}-V_{W}(0))\cdot V_{W}(0):(V_{R}-V_{W}(1))\cdot V_{W}(1):\cdots \\ & :(V_{R}-V_{W}(N-1))\cdot V_{W}(N-1) \end{array}$$

Equation (10) can be used to describe the most critical conclusion to realize the MFBSS algorithm, which shares the transmission bandwidth for the *N*-group scanning of the UPA system. Therefore, according to the ratios of the FIFOs’ lengths, i.e., the cache time of each FIFO, the reading operation can be switched among each FIFO without time slot waiting, thus maximizing the bandwidth utilization ratio.

When the algorithm is implemented by an FPGA, in order to make the consumed resources of the FIFOs minimal, the ratio of *L*(0):*L*(1):…:*L*(*N* − 1) can often be simplified to a series of the suitable integer ratios. In the system of the *N*-group scanning and the *N* FIFOs caches, if the sampling rate *f_sn_* (*n* = 0, 1, …, *N* − 1, and unit is 100 MHz) of the *N* groups linearly increases, *V_R_* = $\sum_{n=0}^{N-1}f_{sn}\cdot \Delta B$, and ΔB = ΔB_W_ = ΔB_R_. The ratios of *L*(0):*L*(1):…:*L*(*N* − 1) of the FIFOs’ lengths are calculated from Equation (10), and the results are listed in Table 2.

**Figure 3:**
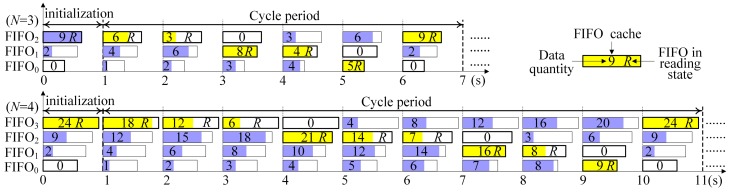
No time-gap switching flow chart of the *N*-group scanning and the *N*-FIFO caches shared.

Figure 3 shows the time slot switching flow chart with the sharing reading bus of the *N*-group scanning and the *N*-FIFO caches (*N* = 3 or 4). The horizontal axis represents the time (unit: s). In the initialization phase, the FIFO*_N_*_−1_ caches the maximum sampling rate beam, which is filled with the length *L*(*N* − 1) data. Meanwhile, the other caches FIFO*_N_*_−2_ ~ FIFO_0_ are filled with the lengths [*L*(*i*) − $\left(\sum_{n=i+1}^{N-1}K_{n}\cdot L(n)\right)/{K^{\prime }}_{i}$](*i* = *N* − 2, *N* − 3, …, 1, 0), respectively. The working principle is described as follows:

When an FIFO is full, it will be immediately read until empty (the symbol R represents the reading state of the FIFO), and subsequently switches to the next FIFO without any time slot in the process of the data transmission. Likewise, when the next FIFO is just written fully, it will be read immediately. Therefore, the whole process is carried out in cycles without any delay, maximizing the utilization ratio of the data transmission bandwidth.

## 4. Implementation and Performance Evaluation of the Scheduling Algorithm

The scheduling algorithm is realized by using a UPA instrument (PA2000 model), which was made by Guangzhou Doppler Electronic Technologies Co., Ltd. (Guangzhou, China), and a Cyclone V GT FPGA Development Board made by Intel Corporation (Santa Clara, CA., USA) as the PCIe communication module with the PC. The UPA data are transmitted to the PC through the PCIe interface, and the multi-group scanning images are processed.

The UPA system with a work clock frequency (*f_s_*) of 100 MHz is mounted with four sensors with four different frequencies (*f_s_*) of 2, 2.5, 5, or 10 MHz, and thus the system can implement 4-group scanning patterns. The echoes of all the groups are up-sampled (*f_s_* = 10 × *f_p_*) by using digital signal processing technology, and thus the actual sampling frequencies of *f_s_*_0_ ~ *f_s_*_3_ become 20, 25, 50, or 100 MHz. The bit-width (ΔB) of the echo data is 8 bits, and both widths of the input (ΔB_W_) and the output (ΔB_R_) ports of the FIFOs are 64 bits. Table 3 lists the parameters of the writing frequency [*V_Wf_*(*n*)] and the reading frequency (*V_Rf_*) of the FIFO*_n_* caches. Obviously, *V_W_*(*n*) equals to *V_Wf_*(*n*) × ΔB_W,_ and *V_R_* equals to *V_Rf_* × ΔB_R_ for this case, hence, the scheduling algorithm can be used to allow the 4-FIFO caches to realize sharing transmission bandwidth. The capacities of the FIFOs are *L*(*n*) × ΔB_W_, and the length ratios of the FIFO caches can be calculated from Equation (10), i.e., *L*(0):*L*(1):*L*(2):*L*(3) = 14:17:29:38. As listed in Table 3, the value of *V_Rf_* is calculated to be 24.375 MHz, but it is relatively easier to implement the value of *V_Rf_* = 25.0 MHz (*V_Rf_* = *f_s_*/4 = 25.0 MHz ≈ *V'_Rf_*) by the FPGA than the value of *V_Rf_* = 24.375 MHz, and thus we design the value of *V_Rf_* = 25.0 MHz for the experiment.

**Figure 4:**
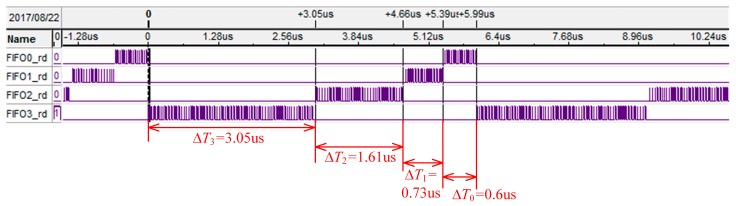
The 4 FIFOs read timing waves of the MFBSS algorithm from Signaltap.

Figure 4 shows the 4 FIFOs reading timing waves of the MFBSS algorithm from Signaltap, and a soft oscilloscope is used to observe FPGA internal signals. The signals of FIFO0_rd ~ FIFO3_rd respectively control the reading operation of the 4 FIFOs, allowing it to enable output data in a time slice polling way. The times for reading the 4 FIFOs until empty are Δ*T*_0_ ~ Δ*T*_3_. The variables of Δ*T*_0_:Δ*T*_1_:Δ*T*_2_:Δ*T*_3_ have the following relation:

$$\Delta T_{0}:\Delta T_{1}:\Delta T_{2}:\Delta T_{3}\approx \frac{L(0)}{V_{Rf}-V_{Wf}(0)}:\frac{L(1)}{V_{Rf}-V_{Wf}(1)}:\frac{L(2)}{V_{Rf}-V_{Wf}(2)}:\frac{L(3)}{V_{Rf}-V_{Wf}(3)}$$

All the FIFOs are readed in turn until empty in every cycle. The sum of data (*D_W−sum_*) for writing into the FIFOs and the sum of data (*D_R−sum_*) for reading out from the FIFOs are given by the two formulas $(\Delta t_{0}\cdot V_{Wf}(0)+\Delta t_{1}\cdot V_{Wf}(1)+$ $\Delta t_{2}\cdot V_{Wf}(2)+\Delta t_{3}\cdot V_{Wf}(3))\cdot \Delta B_{W}$ and $(\Delta t_{0}+\Delta t_{1}+\Delta t_{2}+\Delta t_{3})\cdot V_{Rf}\cdot \Delta B_{W}$, respectively. As a result, the experimental results show that *D_W−sum_* equals to *D_R−sum_*, which meets the relation $V_{R}=\sum_{n=0}^{N-1}V_{W}(n)$ of Equation (2), and also agrees well with the theoretical analysis.

In the *N*-group scanning system, the bandwidth utilization ratio $\eta_{bw}(N)$ of the MFBSS algorithm can be expressed by Equation (11):

(11) $$\eta_{bw}(N)=\frac{\sum_{i=0}^{3}V_{Wf}(i)}{V_{Rf}^{\prime }}\times 100\%.$$

Therefore, in the experiment, when *N* = 4, the utilization ratio $\eta_{bw}(4)$ of the MFBSS algorithm used in the UPA system can be calculated by Equation (12):

(12) $$\eta_{bw}(4)=\frac{\sum_{i=0}^{3}V_{Wf}(i)}{V_{Rf}^{\prime }}\times 100\%=\frac{V_{Rf}}{V_{Rf}^{\prime }}=\frac{24.375}{25}\times 100\%\;=\;97.5\%.$$

The ETSPS scheduling algorithm based on the equal allocation of a time slot to each task. As compared with the MFBSS algorithm in this work, the ETSPS scheduling algorithm has four characteristics: (i) The lengths of all the FIFO*_i_* (*i* = 0, 1, 2, …, *N* − 1) are the same as each other, i.e., *L*(0) = *L*(1) = … = *L*(*N* − 1). (ii) All the time slice resources of the reading operation of the *N* FIFOs are also equal to each other. (iii) All the FIFOs have the reading speed (*V'_R__f_*) which is equal to the maximum of the writing speed [*V_W__f_*(*i*)], same as that of the individual FIFO, i.e., *V'_R__f_* = max[*V_W__f_*(*i*)], *i* = 0, 1, …, *N* − 1. (iv) When the FIFO*_i_* (*i* = 0, 1, 2, …, *N* − 1) is filled by writting, the reading operations of the FIFO*_i_* will be immediately performed. Therfore, the general utilization ratio of the bandwidth-sharing transmission with *N*-group scanning of the UPA system can be calculated by Equation (13):

(13) $$\begin{array}{c} \eta_{bw}^{\prime }(N)=\frac{\sum_{j=0}^{N-1}V_{Wf}(j)}{N\cdot {V^{\prime }}_{Rf}}\times 100\%=\frac{\sum_{j=0}^{N-1}V_{Wf}(j)}{N\cdot \max(V_{Wf}(i))}\times 100\%.\\ i=0,1,\cdot \cdot \cdot N-1 \end{array}$$

For *N*-group scanning data stream with bandwidths {*V_W_*(0), *V_W_*(1), …, *V_W_*(*N* − 1)} (unit: Byte/s), we use the FPGA technology to implement the MFBSS algorithm together with the the traditional ETSPS scheduling algorithm, and analyze their bandwidth utilization ratios $\eta_{bw}(N)$ and $\eta_{bw}^{\prime }(N)$. For example, the FPGA (Arria-II EP2AGX65DF29I5) with a work clock frequency of *f_clk_* = 100 MHz. So, it is easy to produce the clock frequencies such as *F*_1_ = {1, 2, 3, …, *f_clk_* } and *F*_2_ = {*f_clk_*/100, *f_clk_*/99, *f_clk_*/98, …, *f_clk_*/1} (unit: MHz) by using the clock *f_clk_* by Digital Phase Locked Loop technology.

- The MFBSS algorithm. According to Equation (11), the theoretical value of the shared output bandwidth is $V_{Rf}$ or ($\sum_{i=0}^{3}V_{Wf}(i)$). The actual value of the shared output bandwidth is $V_{Rf}^{\prime }$, which satisfies the following conditions: $V_{Rf}^{\prime }\geq V_{Rf}$, $V_{Rf}^{\prime }\in$*F*_1_ or $V_{Rf}^{\prime }\in$*F*_2_, and the value of $\left(V_{Rf}^{\prime }-V_{Rf}\right)$ is minimized. For instance, when $V_{Rf}$ = 24.375 HMz, and $V_{Rf}^{\prime }$ = *f_clk_*/4 = 25 MHz, and thus the actual bandwidth utilization ratio is $\frac{V_{Rf}}{V_{Rf}^{\prime }}\times 100\%$ which equals to 97.5%.
- The ETSPS algorithm. According to Equation (13), the larger the value of max(*V_Wf_*(*i*)) is, the smaller the value of $\eta_{bw}^{\prime }(N)$ is. The smaller the value of max(*V_Wf_*(*i*)) is, the larger the value of $\eta_{bw}^{\prime }(N)$ is. So, when the value of $\max(V_{Wf}(i))$ equals to $\frac{1}{N}\cdot \sum_{j=0}^{N-1}V_{Wf}(j)$, i.e., *V_W_*(0) = *V_W_*(1) = … = *V_W_*(*i*) = … = *V_W_*(*N* − 1), the maximum theoretical value of $\eta_{bw}^{\prime }(N)$ can be expressed by Equation (14).
  (14) $$\max\left(\eta_{bw}^{\prime }(N)\right)=\frac{\sum_{j=0}^{N-1}V_{Wf}(j)}{N\cdot \max(V_{Wf}(i))}\times 100\%=\eta_{bw}(N)$$

when the value of max(*V_Wf_*(*i*)) is close to $\sum_{j=0}^{N-1}V_{Wf}(j)$, i.e., $V_{Wf}(i)\to \sum_{j=0}^{N-1}V_{Wf}(j)$, the minimum theoretical value of $\eta_{bw}^{\prime }(N)$ can be expressed by Equation (15).

(15) $$\min\left(\eta_{bw}^{\prime }(N)\right)\approx \frac{\sum_{j=0}^{N-1}V_{Wf}(j)}{N\cdot \max(V_{Wf}(i))}\times 100\%\approx \left(\frac{100}{N}\right)\%$$

**Figure 5:**
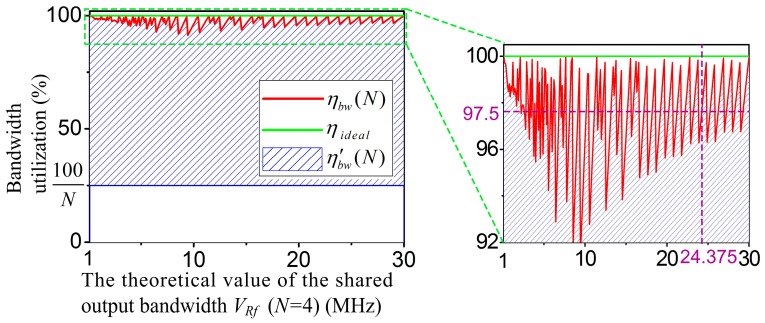
Comparison of the bandwidth utilization ratios of the MFBSS algorithm and the ETSPS algorithm.

Figure 5 shows the bandwidth utilization ratio curves of the two scheduling algorithms (cross axis: the theoretical value of the shared output bandwidth *V_Rf_* (*N* = 4), and vertical axis: the bandwidth utilization). $\eta_{bw}(N)$ and $\eta_{bw}^{\prime }(N)$ are the bandwidth utilization ratios of the MFBSS algorithm and the ETSPS algorithm, respectively.

The symbols $\eta_{bw}(N)$ and $\eta_{ideal}$ represent the experimental and ieal values of the algorithm MFBSS, respectively. The results show that the value of $\eta_{bw}(N)$ is between 92% and 100%, for example, for the above experiment of 4-group scanning based on the MFBSS algorithm, when *V_Rf_* equals to 24.375 MHz, $\eta_{bw}(N)$ equals to 97.5% and $\eta_{ideal}$ equals to 100%. Whereas the value of $\eta_{bw}^{\prime }(N)$ is relevant to the value of *N*, its value is between (100/N)% and $\eta_{bw}(N)$. For *N*-group scanning patterns, only when all groups have the same bandwidth, $\eta_{bw}(N)$ equals to $\eta_{bw}^{\prime }(N)$. Otherwise, $\eta_{bw}^{\prime }(N)$ would be much smaller than $\eta_{bw}(N)$.

**Figure 6:**
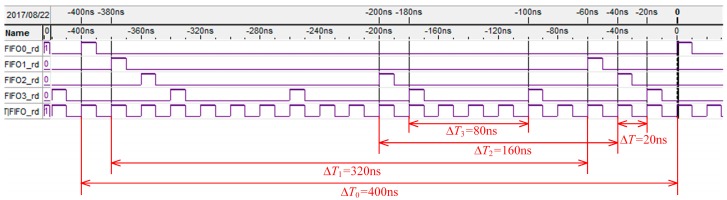
The 4 FIFOs reading timing waves of the ETSPS algorithm from Signaltap.

Similarly, we use FPGA to implement the traditional ETSPS algorithm with the same parameters in Table 3, and collected reading timing waves of the 4 FIFOs by using Signaltap. As shown in Figure 6, the signals FIFO0_rd ~ FIFO3_rd control the reading operation of the four FIFOs, and the time resources occupied by the signals are assigned by the signal FIFO_rd.

Assuming that the symbols *f*_FIFO_rd_, *f*_FIFO0_rd_, *f*_FIFO1_rd_, *f*_FIFO2_rd_, and *f*_FIFO3_rd_ represent the frequencies of signals FIFO_rd, FIFO0_rd, FIFO1_rd, FIFO2_rd, and FIFO3_rd, respectively, the following results can be easily obtained, as shown in Figure 6: *f*_FIFO_rd_ = $\frac{1}{\Delta T}$ = 50 MHz, *f*_FIFO0_rd_ = $\frac{1}{\Delta T_{0}}$ = 2.5 MHz, *f*_FIFO1_rd_ = $\frac{1}{\Delta T_{1}}$= 3.125 MHz, *f*_FIFO2_rd_ = $\frac{1}{\Delta T_{2}}$ = 6.25 MHz, *f*_FIFO3_rd_ =$\frac{1}{\Delta T_{3}}$ = 12.5 MHz.

So, the utilization ratio of the data transmission with the 4-group scanning of the ETSPS algoritnm can be calculated by Equation (16):

(16) $$\begin{array}{cc} \eta_{bw}^{\prime }(4) & =\frac{f_{\mathrm{FIFO}0\_\mathrm{rd}}+f_{\mathrm{FIFO}1\_\mathrm{rd}}+f_{\mathrm{FIFO}2\_\mathrm{rd}}+f_{\mathrm{FIFO}3\_\mathrm{rd}}}{f_{\mathrm{FIFO}\_\mathrm{rd}}}\times 100\%=\frac{\sum_{j=0}^{N-1}f_{sj}}{N\cdot \max(f_{s0},\cdot \cdot \cdot f_{s3})}\times 100\%\\ & =\frac{2.5+3.125+6.25+12.5}{50}\times 100\%\\ & =48.75\% \end{array}$$

As a consequence, the bandwidth utilization ratio of the MFBSS algorithm $\eta_{bw}(4)$ reaches to 97.5% as shown in the inset of Figure 5, while the bandwidth utilization of the ETSPS algorithm $\eta_{bw}^{\prime }(4)$ is only 48.75%. The experimental results demonstrate that the MFBSS algorithm is efficient when used in the multi-group sensors scanning UPA system.

## 5. Conclusions

The novel MFBSS algorithm was proposed on the basis of the FIFOs variable lengths by FPGA technology, and was used for the multi-sensor scanning UPA system to maximize the bandwidth utilization ratio. The mathematical modeling of the MFBSS algorithm was established, and the formula *V_R_* = $\sum_{n=0}^{N-1}V_{W}(n)$ of maximizing bandwidth transmission utilization ratio in the *N*-group scanning patterns was successfully deduced. The lengths of the *N-*group FIFOs were achieved by using the designed equations, from which the length ratios were readily calculated. The algorithm was realized by FPGA technology, which made the reading operation of one FIFO switch to another FIFO without any time slot waiting, and thus it obtained the data transmission bandwidth utilization of no less than 92% hence allowing the UPA system to have the bandwidth utilization higher than that of the traditional ETSPS algorithm. In order to improve transmission efficiency of the large data generated by the sensor systems and the real-time performance of the algorithm through the multi-FPGA technology, the MFBSS scheduling algorithm based on data transmission has important applications in the multi-sensor systems, and the future research is likely to focus on designing some special scheduling algorithm module for different sensor systems.

## Figures and Tables

**Table 1:** The parameters of the *N* groups and the *N* FIFOs caches.

Group Number	Sampling Rate (Hz)	Bit Width	Cache	Length of FIFO	Input Width of FIFO (bit)	Writing Bandwidth (bit/s)	Output Width of FIFO (bit)	Reading Bandwidth (bit/s)
0	*f*_*s*0_	ΔB	FIFO_0_	*L*(0)	ΔB_W_	*V_W_*(0)	ΔB_R_	*V_R_*
1	*f*_*s*1_	ΔB	FIFO_1_	*L*(1)	ΔB_W_	*V_W_*(1)	ΔB_R_	*V_R_*
2	*f*_*s*2_	ΔB	FIFO_2_	*L*(2)	ΔB_W_	*V_W_*(2)	ΔB_R_	*V_R_*
...	...	...	...	...	...	...	...	...
*N* − 1	*f*_*sN*−1_	ΔB	FIFO_*N*−1_	*L*(*N* − 1)	ΔB_W_	*V_W_*(*N* − 1)	ΔB_R_	*V_R_*

**Table 2:** The *N*-group scanning and the *N*-FIFO caches depth ratios.

*N*	*f*_*s*0_	*f*_*s*1_	*f*_*s*2_	*f*_*s*3_	...	*f*_*sN*−1_	*L*(0):*L*(1):...:*L*(*N* − 1)
2	1	2	×	×	×	×	1:1
3	1	2	3	×	×	×	5:8:9
4	1	2	3	4	×	×	9:16:21:24
...	...	...	...	...	...	×	...
*N*	1	2	3	4	...	*N* − 1	(*V_R_* − f_s0_) × *f*_*s*0_:(*V_R_* − *f*_*s*1_) × *f*_*s*1_:...:(*V_R_* − *f*_*sN*−1_) × *f*_*sN*−1_

**Table 3:** The parameters of the 4 groups scanning and the 4 FIFO caches.

f_p_ (MHz)	f_sn_ (MHz)	V_Wf_(n) = f_sn_ × ΔB/ΔBW (MHz)	V_Rf_ = ΣV_Wf_(n) (MHz)	L(n) × ΔB_W_ (bit)
2	20	2.5	24.375	14 × 64
2.5	25	3.125	24.375	17 × 64
5	50	6.25	24.375	29 × 64
10	100	12.5	24.375	38 × 64
